# Association between pre-existing chronic conditions and severity of first SARS-CoV-2 infection symptoms among adults living in Canada: a population-based survey analysis from January 2020 to August 2022

**DOI:** 10.1186/s12889-025-22041-7

**Published:** 2025-03-12

**Authors:** Nicholas Cheta, Dianne Zakaria, Alain Demers, Peri Abdullah, Samina Aziz

**Affiliations:** 1 Lifespan Chronic Diseases and Conditions Division (LCDC), Centre for Surveillance and Applied Research (CSAR), Health Promotion and Chronic Disease Prevention Branch (HPCDP), Public Health Agency of Canada (PHAC), Ottawa, ON Canada; 2 Department of Community Health Sciences, University of Manitoba, Winnipeg, MB Canada

**Keywords:** chronic conditions, SARS-CoV-2, severity, COVID-19, CCAHS-2, multivariable modeling, Canada, cross-sectional survey

## Abstract

**Background:**

Individuals living with chronic conditions (CC) typically have a higher risk of more severe outcomes when exposed to infection. Although many studies have investigated the relationship between CCs and COVID-19 severity, they are generally limited to clinical or hospitalized populations. There is a need to estimate the impact of pre-existing CCs on the severity of acute SARS-CoV-2 infection symptoms among the general population.

**Methods:**

Data from the Canadian COVID-19 Antibody and Health Survey – Cycle 2, a population-based cross-sectional probability survey across 10 provinces capturing the COVID-19 experiences of respondents from January 2020 to August 2022, were used to assess whether pre-existing CCs increased the odds of more severe self-reported infection symptoms among adults living in Canada. Multivariable regression modelling identified which CCs were independently associated with more severe infection symptoms after adjusting for sex, age at infection, and other significant covariates.

**Results:**

Chronic lung disease (aOR = 1.64, 95% CI: 1.09, 2.46), high blood pressure (aOR = 1.35, 95% CI: 1.13, 1.62), weakened immune system (aOR = 1.46, 95% CI: 1.08, 1.98), chronic fatigue syndrome or fibromyalgia (aOR = 2.20, 95% CI: 1.39, 3.50), and arthritis (aOR = 1.28, 95% CI: 1.04, 1.56) were associated with a higher odds of more severe infection, whereas osteoporosis (aOR = 0.58, 95% CI: 0.39, 0.87) was associated with a lower odds. Limiting modelling to adults with confirmed SARS-CoV-2 infections affected some of the variables retained and adjusted associations.

**Conclusion:**

Our findings contribute to a growing evidence base of associations between pre-existing CCs and adverse outcomes after SARS-CoV-2 infection. Identifying factors associated with more severe infection allows for more targeted prevention strategies and early interventions that can minimize the impact of infection.

## Background

Coronavirus disease 2019 (COVID-19), caused by severe acute respiratory syndrome coronavirus 2 (SARS-CoV-2), was declared a global pandemic on March 11, 2020 by the World Health Organization and by March 2023, it was conservatively estimated that around 76% of the Canadian population had been infected [1, 2]. Since the beginning of the outbreak, the daily lives of people have been affected by societal, economic, and public health preventive measures such as use of face masks, physical distancing, quarantines, and lockdowns [3–5].

In addition to the indirect effects of the pandemic, SARS-CoV-2’s acute effect varies widely with respect to the types and severity of symptoms experienced [6]. Among adults in Canada, the most reported acute symptoms are fatigue, fever, coughing, and sore throat, but their severity and impact on daily life is less clear [7]. Those living with chronic conditions (CC) are at a higher risk of more severe outcomes from infectious diseases in general, and the research is growing for SARS-CoV-2 infections specifically [8]. Multiple studies have shown that pre-existing cardiovascular disease (CVD) [9–11], diabetes [12–14], mental illness [15, 16], neurological disease [17–19], musculoskeletal disease [20], and other CCs [21–23] are associated with worse COVID-19 outcomes. Conversely, those with pre-existing respiratory diseases [24–26], fibromyalgia [27], and bowel disease (BD) [28] do not seem to be at increased risk of a more severe infection. However, most studies have only evaluated severe outcomes, like death, in clinical or hospitalized populations, leaving a knowledge gap about the relationship in the general population that experience a SARS-CoV-2 infection without seeking healthcare services. Identifying which chronic conditions put community dwelling adults at increased risk of more severe infections assists in identifying priority populations for targeted prevention strategies (e.g., masking, vaccinations) and early post-infection interventions (e.g., pharmaceuticals).

In addition, existing studies examining the association between pre-existing CCs and severity of a SARS-CoV-2 infection often fail to account for the temporality of important variables. They often are unable to distinguish whether participants had a SARS-CoV-2 infection before or after their CC diagnosis. Additionally, factors such as number of COVID-19 vaccine doses received prior to infection, time since last vaccination prior to infection, and the dominant variant at time of infection are often not considered despite their potential to greatly influence the relationship between pre-existing CCs and the severity of COVID-19.

Cycle 2 of the population-based Canadian COVID-19 Antibody and Health Survey (CCAHS-2) provides a unique opportunity to assess the impact of a SARS-CoV-2 infection on individuals with pre-existing CCs, including those with suspected infections, while accounting for the temporality of key confounders. Using date information provided by the respondents for their vaccines, first SARS-CoV-2 infection, and CC diagnoses, we are able to appropriately sequence events in time and more accurately measure the effect of pre-existing CCs, vaccination status, and dominant variant on severity of SARS-CoV-2 infections. Although other large scale COVID-19 surveys are available, we are unaware of any studies that utilize these data to explore this specific relationship [29–31]. Using CCAHS-2, we aimed to identify pre-existing CCs associated with more severe acute first SARS-CoV-2 infection symptoms in the Canadian adult population self-reporting a confirmed or suspected infection by August 2022.

## Methods

### Study sample and participants

CCAHS-2, a population-based cross-sectional probability survey, was conducted to characterize and estimate the burden of COVID-19 among adults (aged 18 years and older) living in private households across Canada’s 10 provinces. Self-reported information was collected from April to August 2022 using an electronic questionnaire (EQ) specifically developed for the CCAHS-2 through a collaboration between the Public Health Agency of Canada, Statistics Canada, and the COVID-19 Immunity Task Force. Of the 105,998 adults invited to participate, 32,527 (30.7%) completed at least part of the EQ, and 26,859 (25.3%) agreed to share their data with the Public Health Agency of Canada. The response rate for the data used in this study (25.3%) is better than other COVID-19-related national surveys [29–31]. Statistics Canada conducted data validation during and after data collection by comparing both the collected and derived data to comparable Canadian and international data sources to ensure consistency. To mitigate non-response bias, Statistics Canada used characteristics available for both respondents and non-respondents in logistic regression models to identify variables which explained most of the non-response. Variables highly correlated with response or non-response included age group, education, income, census metropolitan area (CMA)/non-CMA, dwelling type, and household size. Based on the modeling results, homogeneous response groups were created and non-response adjustments were applied within these groups to adjust the survey weights. The application of these adjusted weights during analyses helps to minimize non-response bias by accounting for identified differences between respondents and non-respondents. More details about the survey design and the full questionnaire are available on the Statistics Canada website [32]. 

We restricted our analyses to participants that self-reported a confirmed or suspected SARS-CoV-2 infection. By including suspected SARS-CoV-2 infection cases, we were able to account for the population who did not have access to testing or chose not to be tested. A sensitivity analysis was conducted using only participants that self-reported a confirmed SARS-CoV-2 infection.

### Severity of infection

Our outcome of interest, severity of first infection, was captured as follows: no symptoms; mild symptoms – didn’t affect my daily life; moderate symptoms – some effect on my daily life; and severe symptoms – significant effect on my daily life. Using additional information on hospitalization due to symptoms, a trichotomous severity of infection variable was derived (no or mild symptoms, moderate symptoms, severe symptoms or hospitalized) and used as our primary outcome of interest. Respondents who were hospitalized were placed in the highest severity category regardless of their self-reported infection severity. To our knowledge, no validated self-report tool currently exists to measure the severity of SARS-CoV-2 infection symptoms. However, similar four-point scales have been used in other studies assessing COVID-19 symptom severity [33, 34].

### Chronic conditions

Our primary explanatory variables of interest were pre-existing CCs. Participants were asked about 21 different CCs and their dates of diagnosis (year and month if the diagnosis was in 2020 onward and only the year if it was before 2020). CCs were defined as conditions lasting or expected to last at least six months that were diagnosed by a health professional. Rare CCs (liver disease and Alzheimer’s disease or other dementia) were not examined as their frequencies were too low. Additionally, the “other chronic conditions” variable was not examined as the conditions contributing to the category are unknown. However, all three of these CC options were included in the total number of pre-existing CCs covariate. As the survey did not capture date of diagnosis for cancer, it was excluded from the analyses as a pre-existing CC but was considered as a covariate in the regression models. For the remaining CCs, only those diagnosed before a respondent’s first confirmed or suspected SARS-CoV-2 infection were considered pre-existing in the analyses. When only the year of CC diagnosis was provided, and it was the same as the year of reported SARS-CoV-2 infection, the CC status was set to missing as its pre-existence was not establishable. If the month and year of the CC and the infection were the same, it was assumed that the condition existed prior to infection. If the month and the year of the CC were missing, it was assumed that the condition existed prior to infection. We made this assumption for two reasons. First, it is more difficult to recall dates of events occurring in the distant past, so respondents diagnosed in the distant past may not have been able to recall their diagnosis date [35]. Second, it is more likely that the chronic condition was diagnosed prior to infection because of the number of lived years prior to infection compared to the number of lived years between infection and questionnaire completion. Further analysis of our analytic sample substantiated our approach. First, among infected adults, chronic conditions with missing date of diagnosis information were reported by 65 plus-years-olds 63.2% of the time while chronic conditions with date of diagnosis information were reported by 65 plus-years-olds 34.8% of the time (*p* < 0.0001). Second, after excluding chronic conditions with missing date of diagnosis information from our analytic sample, we found that 96.7% of all 18 chronic conditions reported by infected adults were diagnosed prior to SARS-CoV-2 infection. Finally, the proportion of infected adults with a specific chronic condition who did not provide date of diagnosis information never exceeded 3.4% for each of the 18 chronic conditions examined separately. Consequently, the impact of any misclassification resulting from our approach would be minimal. Respondents not completing the CC section of the survey were assumed to have none of the CCs.

### Other explanatory variables

Covariates considered for the regression models include sex at birth, gender, age, sexual orientation, highest household education, ethnicity, dwelling type, place of residence (urban/rural), remoteness index, national and area-based neighbourhood income quintile, Canadian Index of Multiple Deprivation dimensions, region of residence, smoking status, body mass index (BMI), cancer status, number of pre-existing CCs, pre-existing chronic health symptoms (CHSs), number of pre-existing CHSs, disability status, number of COVID-19 vaccine doses before infection, time since last vaccination prior to infection, SARS-CoV-2 testing status, time period of first infection, and household member testing positive for SARS-CoV-2 infection. When the number of respondents with an unknown or missing value for a covariate was 30 or greater with at least 5 respondents in each of the severity of infection categories, an unknown category was defined for the covariate and included in all analyses; otherwise, the missing or unknown data were excluded from all analyses. This approach maximized the number of respondents retained for modeling while ensuring confidentiality requirements were satisfied. Area-based neighbourhood income quintiles are based on a ranking of neighbourhood incomes within each census metropolitan area, census agglomeration and residual neighbourhoods within a province. National neighbourhood income quintiles are based on a ranking of neighbourhood incomes using a national distribution rather than area-based. A community’s index of remoteness is determined by its distance to all population centres defined by Statistics Canada in a given travel radius, as well as their population size [36]. The Canadian Index of Multiple Deprivation dimensions included economic dependency, ethno-cultural composition, residential instability, and situational vulnerability. More information about how these dimensions are defined can be found on the Statistics Canada website [37].

Respondents were asked about 34 CHSs. Similar to the CCs, rare CHSs (fainting, difficulty swallowing, and loss of taste or smell) and “other” CHSs were not specifically examined for reasons previously explained, but were included in the total number of CHSs covariate. As CHSs can result from a SARS-CoV-2 infection, CHSs were considered pre-existing if they first started at least two months prior to the SARS-CoV-2 infection. Otherwise, the pre-existence of each CHS was established using the same approach used for the CCs.

### Analysis

Descriptive statistics include weighted proportions with 95% confidence intervals (CI) calculated using the Clopper-Pearson (exact) method. The design-based first-order Rao-Scott test of association was used to test for group differences (alpha = 0.05, two-tailed).

Multivariable ordinal logistic regression employing complete case analysis was used to determine which CCs were independently associated with severity of infection symptoms. The resulting odds ratio is an estimate of the odds of more severe infection and is assumed to be constant over cumulated lower levels of severity. Considering the large number of variables to be assessed combined with the increased sampling error associated with the analysis of complex survey data, a stepwise selection process was implemented. Briefly, sex at birth, age group at infection, and all CCs were always retained in the model. All other covariates associated with the outcome of interest at an alpha level of 0.10 (two-tailed) during univariable modeling were added one at a time based on the rankings of univariable p-values. If the added covariate was significant at an alpha level of 0.05 (two-tailed) after adjusting for previously selected variables, it was retained, otherwise it was excluded from further consideration. If the addition of a covariate resulted in a previously selected covariate becoming non-significant (*p* > 0.05), the non-significant covariate was permanently removed from the model. This process continued until all initially eligible covariates were assessed. When the main effects model was established, interactions between each retained variable and sex were tested (alpha = 0.05, two-tailed), one at a time, using product terms. Significant interactions were addressed by adding product terms to the final model. Four sensitivity analyses were conducted, repeating the multivariable modelling approach with severity of the first infection as the outcome. The first limited the analytic sample to those reporting a positive polymerase chain reaction or rapid antigen test. The second considered the duration of pre-existing CCs by creating trichotomous CC variables as follows: no CC, CC diagnosed less than 10 years prior to infection, and CC diagnosed 10 or more years prior to infection. The third redefined severity of first infection as a binary variable, distinguishing between adults with severe symptoms or hospitalized and those with moderate, mild, or no symptoms. The last excluded those with missing chronic condition diagnosis dates from the analytic sample to assess any potential bias introduced by our approach to handling missing data. All analyses used sampling and bootstrap weights to account for the complex survey design.

Statistical analyses were performed using SAS version 9.4 (SAS Institute Inc., Cary, NC).

### Ethics approval and consent to participate

This study was exempt from research ethics board review under article 2.2 of the Tri-Council Policy Statement: Ethical Conduct for Research Involving Humans – TCPS 2 (2022) [38]. Our study involved analysis of previously collected anonymized survey data; did not involve linkage to additional data sources; did not include direct follow-up or contact of respondents; and, adhered to the data providers terms and conditions of access, use, and dissemination.

All processes of the CCAHS-2 were reviewed and approved by the Health Canada and Public Health Agency of Canada Research Ethics Board to ensure that internationally recognized ethical standards for human research were met. During the conduct of the survey, adults received invitations by mail that detailed the purpose of the survey and all its components, as well as their right to withdraw from any part of the survey at any time. Completion of the online questionnaire implied consent and captured the respondent’s consent to share their data with specific third parties.

## Results

Table 1 displays the characteristics of adults in Canada self-reporting a first confirmed or suspected infection overall and by severity of infection. Of note, 13 of 18 CCs and nearly all covariates examined were significantly associated with severity of infection. Covariates associated with adults being disproportionately distributed in more severe infection categories included: female sex, female gender, obesity, having a disability, having a suspected infection, being infected prior to the Omicron wave, being unvaccinated or less recently vaccinated prior to infection, reporting any of the specific pre-existing CHSs, and having a greater number of pre-existing chronic conditions or symptoms. Contrarily, having a household member with a confirmed SARS-CoV-2 infection was associated with a less severe infection.

**Table 1:** Characteristics of adults with a self-reported confirmed or suspected SARS-CoV-2 infection by severity of first infection symptoms, Canada, January 2020 to August 2022

	All *N* = 9132		No or Mild Symptoms *N* = 3508		Moderate Symptoms *N* = 4000		Severe Symptoms or Hospitalized *N* = 1624		*P* value
Characteristics	Percent	95% CI	Percent	95% CI	Percent	95% CI	Percent	95% CI	
Sex at birth									< 0.001
male	48.1	(47.1, 49.1)	54.1	(51.9, 56.2)	45.2	(43.3, 47.1)	42.1	(38.9, 45.3)	
female	51.9	(50.9, 52.9)	45.9	(43.8, 48.1)	54.8	(52.9, 56.7)	57.9	(54.7, 61.1)	
Gender									< 0.001
men	48.0	(46.9, 49.0)	53.8	(51.7, 55.9)	45.1	(43.2, 47.1)	41.9	(38.7, 45.1)	
women	51.7	(50.6, 52.7)	45.7	(43.6, 47.8)	54.7	(52.7, 56.6)	57.5	(54.3, 60.7)	
other	0.4	(0.2, 0.6)	-^a^	-	-	-	-	-	
Age at infection									0.002
15–34	36.4	(35.4, 37.5)	35.9	(33.8, 37.9)	38.2	(36.4, 40.2)	33.3	(30.2, 36.6)	
35–49	30.0	(29.1, 31.0)	29.7	(28.0, 31.6)	30.3	(28.6, 32.0)	29.8	(27.0, 32.7)	
50–64	22.5	(21.6, 23.3)	21.7	(20.2, 23.3)	22.0	(20.6, 23.5)	25.3	(22.7, 28.0)	
65+	11.1	(10.4, 11.7)	12.7	(11.6, 13.8)	9.4	(8.5, 10.4)	11.6	(9.8, 13.5)	
Sexual orientation									0.548
heterosexual	92.5	(91.7, 93.3)	93.1	(91.6, 94.3)	92.1	(90.8, 93.3)	92.4	(90.5, 94.0)	
homosexual	2.5	(2.0, 3.0)	1.9	(1.2, 2.7)	3.0	(2.2, 3.9)	2.5	(1.6, 3.7)	
other (bisexual, sapiosexual, etc…)	3.5	(3.0, 4.1)	3.6	(2.7, 4.7)	3.5	(2.7, 4.3)	3.7	(2.5, 5.1)	
unknown	1.5	(1.1, 1.9)	1.5	(1.0, 2.2)	1.4	(0.9, 2.1)	1.4	(0.8, 2.4)	
Highest education completed in household									0.163
less than secondary school graduation	2.6	(2.2, 3.0)	3.0	(2.4, 3.8)	2.1	(1.6, 2.8)	2.9	(2.0, 4.1)	
secondary school graduation	10.6	(9.8, 11.5)	11.1	(9.8, 12.5)	10.0	(8.9, 11.3)	11.0	(9.2, 13.1)	
post-secondary school graduation	86.8	(85.9, 87.7)	85.9	(84.4, 87.3)	87.9	(86.5, 89.1)	86.0	(83.7, 88.2)	
Ethnicity									< 0.001
Indigenous	3.1	(2.6, 3.5)	2.5	(2.0, 3.1)	3.3	(2.7, 4.1)	3.7	(2.6, 5.0)	
White	72.7	(71.3, 74.2)	70.3	(67.9, 72.6)	74.5	(72.4, 76.5)	73.9	(70.5, 77.0)	
South Asian	5.7	(4.9, 6.5)	5.9	(4.6, 7.3)	5.2	(4.1, 6.5)	6.4	(4.5, 8.9)	
East/Southeast Asian	8.2	(7.3, 9.1)	10.1	(8.5, 11.9)	7.7	(6.4, 9.1)	5.1	(3.6, 6.9)	
Black	2.6	(2.1, 3.1)	2.9	(2.1, 3.8)	2.1	(1.5, 2.9)	3.1	(1.7, 5.0)	
Arab/West Asian	3.1	(2.6, 3.7)	4.0	(3.0, 5.2)	2.0	(1.5, 2.7)	3.8	(2.5, 5.4)	
Latin American	2.2	(1.7, 2.6)	2.3	(1.6, 3.3)	2.0	(1.4, 2.7)	2.2	(1.3, 3.5)	
Mixed/Other	2.5	(2.0, 3.0)	2.0	(1.4, 2.8)	3.2	(2.3, 4.2)	1.9	(1.1, 3.0)	
Dwelling type									0.09
single detached, double, or duplex	71.3	(69.9, 72.7)	71.1	(68.9, 73.2)	71.6	(69.6, 73.5)	70.8	(67.4, 74.1)	
row, terrace, or low-rise apartment	19.2	(18.1, 20.4)	19.1	(17.3, 21.0)	20.1	(18.4, 21.7)	17.2	(14.5, 20.1)	
high-rise apartment	8.1	(7.2, 9.1)	8.2	(6.8, 9.9)	7.3	(6.0, 8.7)	10.1	(7.9, 12.7)	
other (institution, hotel, etc…)	1.4	(1.1, 1.7)	1.5	(1.1, 2.1)	1.1	(0.7, 1.5)	1.9	(1.0, 3.3)	
Urban/rural residence									0.056
urban	85.7	(84.9, 86.5)	84.5	(83.1, 85.9)	86.2	(84.9, 87.4)	87.3	(85.2, 89.2)	
rural	14.3	(13.5, 15.1)	15.5	(14.1, 16.9)	13.8	(12.6, 15.1)	12.7	(10.8, 14.8)	
Remoteness index									< 0.001
easily accessible area	78.0	(77.1, 78.8)	76.9	(75.3, 78.3)	79.5	(78.2, 80.8)	76.6	(74.2, 78.9)	
accessible area	17.1	(16.3, 17.8)	18.0	(16.7, 19.3)	15.4	(14.3, 16.6)	19.1	(17.1, 21.3)	
less accessible area	4.0	(3.6, 4.4)	3.8	(3.2, 4.5)	4.4	(3.8, 5.1)	3.5	(2.6, 4.5)	
remote or very remote area	0.6	(0.4, 0.8)	0.9	(0.6, 1.3)	0.3	(0.2, 0.6)	-	-	
unknown	0.4	(0.3, 0.5)	0.5	(0.3, 0.8)	0.3	(0.2, 0.5)	-	-	
National neighbourhood income quintile before tax									0.148
first (lowest)	16.2	(15.3, 17.1)	17.8	(16.2, 19.5)	15.0	(13.6, 16.3)	15.7	(13.7, 17.8)	
second	19.4	(18.3, 20.5)	18.5	(16.8, 20.3)	20.0	(18.4, 21.7)	19.7	(17.3, 22.3)	
third	20.9	(19.8, 22.0)	21.0	(19.2, 22.9)	20.7	(19.1, 22.4)	21.0	(18.3, 23.9)	
fourth	21.6	(20.4, 22.8)	21.3	(19.5, 23.3)	21.1	(19.3, 22.9)	23.4	(20.6, 26.5)	
fifth (highest)	22.0	(20.8, 23.1)	21.4	(19.5, 23.3)	23.2	(21.5, 25.0)	20.2	(17.7, 22.8)	
Area-based neighbourhood income quintile before tax									0.041
first (lowest)	16.4	(15.4, 17.5)	17.0	(15.3, 18.9)	15.7	(14.3, 17.2)	16.9	(14.7, 19.3)	
second	19.6	(18.7, 20.9)	20.4	(18.6, 22.4)	18.2	(16.6, 19.8)	21.6	(18.9, 24.5)	
third	20.7	(19.5, 21.8)	18.7	(16.9, 20.5)	22.5	(20.8, 24.2)	20.5	(17.7, 23.5)	
fourth	21.6	(20.3, 22.6)	21.8	(20.1, 23.7)	21.4	(19.7, 23.2)	21.6	(18.9, 24.6)	
fifth (highest)	21.7	(20.5, 22.8)	22.0	(20.2, 23.9)	22.2	(20.6, 24.0)	19.4	(16.9, 22.1)	
Economic dependency quintile^b^									0.221
first (lowest)	24.3	(23.1, 25.6)	24.0	(22.0, 26.1)	24.5	(22.7, 26.4)	24.5	(21.7, 27.6)	
second	20.5	(19.4, 21.7)	19.2	(17.4, 21.1)	21.9	(20.1, 23.8)	20.1	(17.3, 23.2)	
third	17.5	(16.5, 18.6)	18.7	(16.9, 20.6)	17.3	(15.7, 18.9)	15.6	(13.3, 18.2)	
fourth	14.8	(13.8, 15.7)	15.7	(14.2, 17.4)	14.1	(12.7, 15.6)	14.3	(12.2, 16.6)	
fifth (highest)	13.7	(12.8, 14.6)	13.6	(12.2, 15.1)	13.4	(12.1, 14.8)	14.7	(12.6, 17.0)	
unknown	9.1	(8.4, 9.9)	8.8	(7.6, 10.1)	8.8	(7.7, 10.0)	10.7	(8.7, 12.9)	
Ethno-cultural composition quintile^c^									< 0.001
first (lowest)	14.3	(13.5, 15.1)	16.3	(14.9, 17.8)	13.4	(12.2, 14.7)	12.2	(10.5, 14.1)	
second	15.3	(14.4, 16.3)	15.5	(14.0, 17.1)	14.5	(13.1, 15.9)	17.0	(14.7, 19.5)	
third	18.5	(17.4, 19.5)	18.5	(16.7, 20.3)	18.9	(17.4, 20.6)	17.3	(15.0, 19.7)	
fourth	22.1	(20.9, 23.3)	19.4	(17.6, 21.3)	24.8	(22.9, 26.7)	21.4	(18.7, 24.4)	
fifth (highest)	20.7	(19.4, 21.9)	21.5	(19.4, 23.7)	19.6	(17.7, 21.6)	21.4	(18.2, 25.0)	
unknown	9.1	(8.4, 9.9)	8.8	(7.6, 10.1)	8.8	(7.7, 10.0)	10.7	(8.7, 12.9)	
Residential instability quintile^d^									0.235
first (lowest)	15.5	(14.5, 16.5)	16.5	(14.9, 18.2)	15.3	(13.9, 16.7)	13.9	(11.8, 16.3)	
second	17.9	(16.9, 19.0)	18.1	(16.4, 19.8)	18.6	(17.0, 20.2)	16.0	(13.7, 18.6)	
third	18.3	(17.2, 19.4)	18.7	(16.9, 20.5)	17.4	(15.9, 18.9)	19.8	(17.0, 22.7)	
fourth	18.0	(16.9, 19.1)	17.7	(16.0, 19.5)	18.6	(17.0, 20.3)	17.2	(14.8, 19.9)	
fifth (highest)	21.1	(19.9, 22.4)	20.2	(18.3, 22.3)	21.4	(19.7, 23.3)	22.4	(19.6, 25.3)	
unknown	9.1	(8.4, 9.9)	8.8	(7.6, 10.1)	8.8	(7.7, 10.0)	10.7	(8.7, 12.9)	
Situational vulnerability quintile^e^									0.04
first (lowest)	24.8	(23.5, 26.1)	24.4	(22.4, 26.4)	25.1	(23.2, 27.1)	24.7	(21.9, 27.8)	
second	19.9	(18.8, 21.0)	20.0	(18.2, 21.9)	20.1	(18.4, 21.9)	19.0	(16.4, 21.7)	
third	18.7	(17.7, 19.7)	18.2	(16.5, 20.0)	19.2	(17.5, 20.9)	18.8	(16.1, 21.6)	
fourth	15.5	(14.5, 16.5)	14.5	(13.0, 16.0)	16.3	(14.9, 17.9)	15.6	(13.2, 18.2)	
fifth (highest)	12.1	(11.2, 12.9)	14.2	(12.7, 15.8)	10.5	(9.3, 11.7)	11.2	(9.5, 13.2)	
unknown	9.1	(8.4, 9.9)	8.8	(7.6, 10.1)	8.8	(7.7, 10.0)	10.7	(8.7, 12.9)	
Province of Residence									< 0.001
British Columbia	14.0	(13.3, 14.7)	12.1	(10.8, 13.4)	14.7	(13.5, 15.9)	16.3	(14.2, 18.6)	
Prairies	19.2	(18.4, 20.0)	18.7	(17.4, 20.1)	20.0	(18.7, 21.2)	18.3	(16.3, 20.6)	
Ontario	37.3	(36.1, 38.4)	37.7	(35.6, 39.9)	36.0	(34.1, 38.1)	39.3	(35.8, 42.8)	
Quebec	23.1	(22.3, 24.0)	24.6	(23.0, 26.2)	23.3	(21.8, 24.8)	19.6	(17.3, 22.0)	
Atlantic Canada	6.4	(5.9, 7.0)	6.9	(6.1, 7.8)	6.0	(5.3, 6.8)	6.5	(5.3, 7.8)	
Smoking status									0.513
current smoker	8.2	(7.5, 8.9)	8.3	(7.1, 9.5)	7.8	(6.7, 8.9)	9.0	(7.3, 10.9)	
does not currently smoke	91.8	(91.1, 92.5)	91.7	(90.5, 92.9)	92.2	(91.1, 93.3)	91.0	(89.1, 92.7)	
Body mass index (BMI)									< 0.001
underweight and normal weight (BMI < 25 kg/m2)	31.3	(30.0, 32.5)	32.7	(30.5, 34.9)	31.7	(29.7, 33.7)	27.1	(24.1, 30.2)	
overweight (25 kg/m2 < = BMI < 30 kg/m2)	35.7	(34.4, 37.0)	36.4	(34.3, 38.6)	37.0	(35.0, 39.1)	30.9	(27.8, 34.1)	
obese (BMI > = 30 kg/m2)	30.9	(29.7, 32.1)	29.1	(27.1, 31.0)	28.9	(27.2, 30.7)	39.8	(36.5, 43.3)	
unknown	2.2	(1.8, 2.6)	1.9	(1.3, 2.6)	2.4	(1.8, 3.1)	2.2	(1.3, 3.5)	
Cancer									0.288
current cancer	1.4	(1.2, 1.8)	1.2	(0.9, 1.7)	1.4	(1.0, 2.0)	1.9	(1.2, 2.8)	
does not currently have cancer	98.6	(98.2, 98.8)	98.8	(98.3, 99.1)	98.6	(98.0, 99.0)	98.1	(97.2, 98.8)	
Pre-existing chronic conditions									
chronic lung disease	1.6	(1.3, 1.9)	1.1	(0.8, 1.5)	1.4	(1.0, 1.8)	3.5	(2.5, 4.7)	< 0.001
sleep apnea	5.7	(5.1, 6.3)	4.8	(4.0, 5.7)	5.6	(4.7, 6.5)	7.9	(6.4, 9.7)	0.001
asthma	7.5	(6.9, 8.2)	6.1	(5.1, 7.2)	7.2	(6.2, 8.3)	11.6	(9.6, 13.9)	< 0.001
chronic heart disease	2.1	(1.7, 2.4)	2.0	(1.6, 2.6)	1.8	(1.4, 2.4)	2.7	(1.9, 3.9)	0.194
diabetes	5.2	(4.7, 5.8)	5.6	(4.7, 6.8)	3.8	(3.2, 4.5)	7.9	(6.3, 9.6)	< 0.001
chronic kidney disease	0.6	(0.4, 0.9)	0.5	(0.2, 0.9)	0.8	(0.5, 1.3)	0.4	(0.2, 0.9)	0.176
high blood pressure	11.9	(11.1, 12.7)	10.8	(9.7, 12.1)	10.8	(9.8, 11.9)	17.1	(14.7, 19.6)	< 0.001
chronic blood disorder	0.8	(0.6, 1.0)	0.8	(0.4, 1.3)	0.6	(0.4, 0.9)	1.2	(0.6, 2.0)	0.158
osteoporosis	1.9	(1.6, 2.2)	2.1	(1.5, 2.7)	1.3	(1.0, 1.8)	2.8	(2.0, 3.9)	0.004
back problems	10.5	(9.7, 11.2)	8.9	(7.7, 10.2)	9.2	(8.2, 10.2)	17.3	(15.0, 19.8)	< 0.001
urinary incontinence	1.9	(1.6, 2.3)	1.7	(1.2, 2.2)	1.9	(1.5, 2.5)	2.6	(1.8, 3.6)	0.176
bowel disorder	4.1	(3.6, 4.7)	3.4	(2.7, 4.3)	3.5	(2.8, 4.3)	7.3	(5.8, 9.0)	< 0.001
weakened immune system	3.7	(3.2, 4.2)	2.3	(1.7, 3.0)	3.4	(2.7, 4.3)	7.3	(5.6, 9.2)	< 0.001
chronic neurological disorder	1.7	(1.4, 2.0)	1.4	(0.9, 2.0)	1.4	(1.0, 1.9)	3.1	(2.1, 4.3)	< 0.001
chronic fatigue syndrome or fibromyalgia	1.4	(1.2, 1.7)	0.6	(0.3, 0.9)	1.2	(0.8, 1.6)	4.1	(3.0, 5.5)	< 0.001
effects of a stroke	0.5	(0.3, 0.7)	0.5	(0.3, 0.8)	0.5	(0.2, 0.8)	0.6	(0.3, 1.1)	0.89
mental health condition	11.5	(10.6, 12.3)	9.2	(7.9, 10.7)	10.9	(9.6, 12.3)	17.9	(15.6, 20.3)	< 0.001
arthritis	10.8	(10.1, 11.6)	8.9	(7.9, 10.0)	9.7	(8.7, 10.9)	18	(15.7, 20.5)	< 0.001
Number of pre-existing chronic conditions									< 0.001
0	55.7	(54.3, 57.0)	59.0	(56.8, 61.1)	58.3	(56.2, 60.4)	41.6	(38.2, 45.0)	
1	22.6	(21.5, 23.8)	23.3	(21.5, 25.2)	22.4	(20.6, 24.2)	21.8	(19.2, 24.6)	
2	11.4	(10.6, 12.2)	9.5	(8.3, 10.8)	10.3	(9.2, 11.4)	18.4	(15.9, 21.2)	
3	5.0	(4.5, 5.6)	4.5	(3.7, 5.4)	4.4	(3.7, 5.1)	7.9	(6.4, 9.6)	
> 3	5.2	(4.7, 5.8)	3.7	(3.0, 4.4)	4.6	(3.9, 5.5)	10.3	(8.7, 12.1)	
Pre-existing chronic symptoms									
pain (excluding headache)	12.9	(12.1, 13.7)	10.3	(9.2, 11.6)	12.2	(11.0, 13.5)	20.2	(17.7, 22.8)	< 0.001
shortness of breath or difficulty breathing	4.8	(4.2, 5.3)	4.1	(3.3, 5.0)	4.2	(3.3, 5.0)	7.6	(6.1, 9.3)	< 0.001
difficulty speaking or hoarseness	0.7	(0.6, 0.9)	0.6	(0.4, 0.9)	0.4	(0.2, 0.6)	1.9	(1.2, 2.7)	< 0.001
cough	3.0	(2.6, 3.4)	2.5	(1.9, 3.1)	2.7	(2.1, 3.3)	5.0	(3.8, 6.4)	< 0.001
headache	6.9	(6.2, 7.6)	4.9	(4.0, 5.9)	6.9	(5.9, 8.0)	11.2	(9.3, 13.3)	< 0.001
chest tightness	1.8	(1.5, 2.2)	1.5	(1.0, 2.0)	1.6	(1.1, 2.3)	3.1	(2.2, 4.3)	0.003
symptoms relating to the heart (e.g., fast, pounding or irregular heartbeat)	4.3	(3.8, 4.8)	3.0	(2.4, 3.7)	4.1	(3.4, 5.0)	7.4	(6.0, 9.2)	< 0.001
fatigue, tiredness or loss of energy	13.5	(12.6, 14.5)	9.4	(8.3, 10.7)	13.8	(12.3, 15.3)	22.1	(19.4, 24.9)	< 0.001
general weakness	3.4	(2.9, 3.9)	1.7	(1.3, 2.3)	3.7	(3.0, 4.6)	6.1	(4.7, 7.7)	< 0.001
loss of appetite	1.2	(0.9, 1.5)	0.7	(0.4, 1.0)	0.9	(0.6, 1.4)	2.9	(2.0, 4.0)	< 0.001
feeling thirsty	2.2	(1.9, 2.6)	1.9	(1.3, 2.6)	1.9	(1.5, 2.5)	3.8	(2.8, 4.9)	< 0.001
nausea, vomiting	1.4	(1.1, 1.8)	1.1	(0.6, 1.6)	1.2	(0.7, 1.9)	2.6	(1.8, 3.6)	0.005
upset stomach, bloating, gas	7.2	(6.5, 7.9)	5.4	(4.5, 6.4)	7.3	(6.2, 8.5)	11	(9.1, 13.1)	< 0.001
heartburn or indigestion	7.2	(6.6, 7.8)	5.2	(4.4, 6.1)	7.7	(6.7, 8.8)	10.4	(8.6, 12.5)	< 0.001
frequent urination	5.1	(4.6, 5.6)	4.5	(3.8, 5.3)	4.5	(3.8, 5.3)	7.7	(6.2, 9.5)	< 0.001
irregular bowel movements or habits	7.0	(6.4, 7.7)	5.1	(4.2, 6.1)	7.3	(6.2, 8.5)	10.6	(8.8, 12.7)	< 0.001
change in body weight	2.8	(2.4, 3.2)	2.4	(1.8, 3.2)	2.3	(1.8, 2.9)	4.6	(3.4, 5.9)	< 0.001
dizziness	3.8	(3.3, 4.3)	2.7	(2.0, 3.5)	3.5	(2.8, 4.3)	6.9	(5.3, 8.7)	< 0.001
feeling hot or cold (body temperature changes)	5	(4.4, 5.5)	3.4	(2.8, 4.1)	4.7	(3.9, 5.6)	9.2	(7.6, 11.1)	< 0.001
numbness or tingling	5.1	(4.6, 5.7)	3.4	(2.8, 4.1)	5.3	(4.4, 6.3)	8.7	(7.1, 10.5)	< 0.001
swelling	2.3	(2.0, 2.7)	1.7	(1.3, 2.3)	1.8	(1.3, 2.3)	5.2	(4.0, 6.8)	< 0.001
skin irritation	7.2	(6.5, 7.9)	5.4	(4.5, 6.4)	7.6	(6.5, 8.7)	10.4	(8.3, 12.7)	< 0.001
joint inflammation	10	(9.2, 10.7)	7.5	(6.6, 8.6)	9.2	(8.1, 10.4)	17.4	(15.1, 19.8)	< 0.001
stiffness	9.4	(8.7, 10.2)	7.4	(6.4, 8.5)	9.2	(8.1, 10.5)	14.5	(12.5, 16.7)	< 0.001
difficulty falling or staying asleep	12.3	(11.5, 13.1)	9.8	(8.6, 11.1)	12.5	(11.3, 13.8)	17.2	(15.1, 19.6)	< 0.001
difficulty thinking or problem solving (brain fog)	5.7	(5.0, 6.3)	3.9	(3.1, 4.9)	5.9	(5.0, 7.0)	8.8	(7.1, 10.8)	< 0.001
confusion, memory loss	3.2	(2.7, 3.6)	2.4	(1.8, 3.1)	2.7	(2.2, 3.4)	5.9	(4.4, 7.6)	< 0.001
loss of interest in activities	5.4	(4.9, 6.0)	4.4	(3.6, 5.3)	5.1	(4.3, 6.1)	8.6	(7.0, 10.4)	< 0.001
sadness, pessimism, hopelessness, or depression	9.1	(8.3, 9.9)	7.9	(6.7, 9.2)	8.2	(7.1, 9.4)	13.9	(11.8, 16.3)	< 0.001
stress or anxiety	22.2	(21.0, 23.4)	18.8	(17.0, 20.7)	21.6	(19.9, 23.3)	31.5	(28.4, 34.7)	< 0.001
Number of pre-existing chronic health symptoms									< 0.001
0	49.9	(48.5, 51.3)	55.8	(53.5, 58.0)	50.1	(48.1, 52.2)	35.9	(32.6, 39.3)	
1–2	25.8	(24.6, 27.1)	25.6	(23.7, 27.7)	25.7	(23.9, 27.6)	26.4	(23.5, 29.5)	
3	6.5	(5.9, 7.2)	5.1	(4.2, 6.2)	7.2	(6.2, 8.3)	7.9	(6.3, 9.7)	
> 3	17.8	(16.9, 18.8)	13.4	(12.1, 14.9)	17.0	(15.6, 18.4)	29.8	(27.0, 32.7)	
Disability status									< 0.001
identifies as having a disability	6.0	(5.5, 6.7)	5.2	(4.3, 6.3)	5.2	(4.4, 6.2)	9.9	(8.4, 11.7)	
does not identify as having a disability	92.3	(91.6, 93.0)	93.7	(92.5, 94.7)	92.6	(91.4, 93.7)	88.6	(86.7, 90.2)	
unknown	1.6	(1.3, 2.1)	1.1	(0.6, 1.8)	2.2	(1.5, 3.0)	1.5	(1.0, 2.2)	
Number of vaccine doses received prior to the month of infection									< 0.001
0	24.3	(22.9, 25.7)	20.5	(18.4, 22.7)	22.1	(20.2, 24.1)	38.2	(34.9, 41.6)	
1	2.0	(1.6, 2.4)	2.0	(1.4, 2.8)	1.9	(1.4, 2.6)	2.0	(1.0, 3.6)	
2	35.1	(33.8, 36.5)	35.8	(33.4, 38.1)	35.8	(33.9, 37.9)	31.9	(28.8, 35.1)	
> 2	36.6	(35.2, 38.0)	39.4	(37.2, 41.8)	38.3	(36.3, 40.4)	25.8	(23.0, 28.7)	
unknown	2.0	(1.7, 2.4)	2.3	(1.7, 2.9)	1.8	(1.3, 2.5)	2.1	(1.2, 3.4)	
Months since last vaccine dose prior to the month of infection									< 0.001
not vaccinated	24.2	(22.9, 25.7)	20.5	(18.4, 22.7)	22.1	(20.2, 24.1)	38.2	(34.9, 41.6)	
<=3 months	25.3	(24.1, 26.5)	28.6	(26.6, 30.6)	25.4	(23.5, 27.3)	17.6	(15.2, 20.3)	
4–6 months	33.7	(32.3, 35.0)	33.1	(30.9, 35.4)	36.7	(34.7, 38.8)	27.3	(24.3, 30.5)	
> 6 months	14.4	(13.4, 15.3)	15.1	(13.4, 16.8)	13.7	(12.3, 15.2)	14.3	(12.0, 16.9)	
unknown	2.4	(2.1, 2.9)	2.8	(2.2, 3.5)	2.1	(1.5, 2.8)	2.5	(1.6, 3.9)	
SARS-CoV-2 testing status									< 0.001
confirmed infection (PCR or RAT positive)	76.1	(74.8, 77.3)	78.2	(76.3, 80.1)	77.3	(75.5, 79.0)	68.1	(64.9, 71.1)	
suspected infection	23.9	(22.7, 25.2)	21.8	(19.9, 23.7)	22.7	(21.0, 23.7)	31.9	(28.9, 35.1)	
Time period of first infection									< 0.001
pre-Omicron (January 1, 2020 – November 30, 2021)	24.2	(22.9, 25.5)	20.4	(18.5, 22.4)	21.7	(19.9, 23.6)	39.0	(35.7, 42.4)	
Omicron (December 1, 2021 – August 31, 2022)	75.1	(73.8, 76.4)	79.0	(77.0, 80.9)	77.9	(76.0, 79.7)	59.6	(56.2, 62.9)	
unknown	0.7	(0.5, 1.0)	0.6	(0.3, 1.1)	0.4	(0.2, 0.7)	-	-	
Household member testing positive for a SARS-CoV-2 infection									< 0.001
yes	61.7	(60.1, 63.1)	65.3	(62.9, 67.5)	61.1	(59.0, 63.3)	54.8	(51.3, 58.4)	
no	37.4	(35.9, 38.9)	34.1	(31.8, 36.4)	37.9	(35.8, 40.1)	43.7	(40.2, 47.3)	
unknown	0.9	(0.7, 1.3)	0.7	(0.4, 1.1)	1.0	(0.5, 1.6)	-	-	

Notes: Estimates for Canada exclude the territories. All estimates are weighted

Abbreviations: CI = confidence interval, PCR = polymerase chain reaction, RAT = rapid antigen test

^a^ Suppressed as coefficient of variation is greater than 33.3%

^b^ Economic dependency quantifies a neighbourhood’s dependence on sources of income other than employment income

^c^ Ethno-cultural composition quantifies a neighbourhood’s makeup of immigrant populations

^d^ Residential instability quantifies the tendency of neighbourhood inhabitants to fluctuate over time

^e^ Situational vulnerability quantifies a neighbourhood’s education level, Indigenous composition, and extent of dwellings in need of major repairs

In the fully adjusted model, 6 of the 18 pre-existing CCs remained significantly associated with severity of infection after adjusting for other important covariates. Pre-existing chronic lung disease (CLC) (adjusted odds ratio (aOR): 1.64, 95% CI: 1.09, 2.46), high blood pressure (HBP) (aOR: 1.35, 95% CI: 1.13, 1.62), weakened immune system (WIS) (aOR: 1.46, 95% CI: 1.08, 1.98), chronic fatigue syndrome (CFS) or fibromyalgia (aOR: 2.20, 95% CI: 1.39, 3.50), and arthritis (aOR: 1.28, 95% CI: 1.04, 1.56) were associated with higher odds of more severe infection (Table 2). Conversely, pre-existing osteoporosis was associated with lower odds of a more severe infection (aOR: 0.58, 95% CI: 0.39, 0.87).

**Table 2:** Odds ratios for more severe acute first infection symptoms among adults with a self-reported confirmed or suspected SARS-CoV-2 infection, Canada, January 2020 to August 2022 (*n* = 9086)

Characteristics	uOR	95% CI	aOR	95% CI	*p* value*
Pre-existing chronic conditions					
chronic lung disease	2.41	(1.67, 3.48)	1.64	(1.09, 2.46)	0.017
sleep apnea	1.42	(1.14, 1.76)	1.17	(0.93, 1.49)	0.186
asthma	1.59	(1.29, 1.95)	1.22	(0.98, 1.52)	0.072
chronic heart disease	1.17	(0.83, 1.64)	0.88	(0.61, 1.27)	0.502
diabetes	1.12	(0.85, 1.46)	0.87	(0.66, 1.17)	0.360
chronic kidney disease	1.13	(0.71, 1.80)	0.95	(0.55, 1.65)	0.865
high blood pressure	1.36	(1.16, 1.59)	1.35	(1.13, 1.62)	< 0.001
chronic blood disorder	1.18	(0.59, 2.36)	0.66	(0.30, 1.45)	0.299
osteoporosis	1.08	(0.72, 1.62)	0.58	(0.39, 0.87)	0.009
back problems	1.62	(1.36, 1.93)	1.11	(0.91, 1.36)	0.321
urinary incontinence	1.33	(0.96, 1.85)	0.91	(0.65, 1.27)	0.563
bowel disorder	1.71	(1.29, 2.26)	1.06	(0.81, 1.40)	0.658
weakened immune system	2.35	(1.76, 3.12)	1.46	(1.08, 1.98)	0.015
chronic neurological disorder	1.73	(1.10, 2.70)	0.94	(0.60, 1.49)	0.801
chronic fatigue syndrome or fibromyalgia	4.57	(3.00, 6.97)	2.20	(1.39, 3.50)	< 0.001
stroke	1.06	(0.58, 1.94)	0.77	(0.40, 1.50)	0.446
mental health condition	1.64	(1.39, 1.95)	1.12	(0.93, 1.35)	0.238
arthritis	1.7	(1.44, 2.00)	1.28	(1.04, 1.56)	0.019
Age at infection (ref = 15–34)	-	-	-	-	< 0.001
35–49	1.02	(0.90, 1.16)	1.04	(0.91, 1.19)	0.540
50–64	1.10	(0.96, 1.26)	0.96	(0.83, 1.12)	0.632
65+	0.87	(0.74, 1.02)	0.66	(0.54, 0.81)	< 0.001
Sex (ref = male)	-	-	-	-	< 0.001
female	1.44	(1.29, 1.60)	1.30	(1.15, 1.46)	< 0.001
Ethnicity (ref = white)	-	-	-	-	0.023
Indigenous	1.26	(0.97, 1.64)	1.10	(0.84, 1.46)	0.487
South Asian	0.97	(0.71, 1.32)	0.94	(0.68, 1.31)	0.715
East/Southeast Asian	0.64	(0.51, 0.80)	0.63	(0.49, 0.80)	< 0.001
Black	0.90	(0.57, 1.44)	0.81	(0.50, 1.31)	0.387
Arab/West Asian	0.73	(0.48, 1.10)	0.73	(0.47, 1.15)	0.176
Latin American	0.87	(0.56, 1.34)	0.89	(0.58, 1.38)	0.604
Mixed/Other	1.09	(0.80, 1.47)	1.09	(0.79, 1.51)	0.600
Ethno-cultural composition (ref = first quintile)^a^	-	-	-	-	0.001
second	1.27	(1.07, 1.51)	1.26	(1.05, 1.52)	0.013
third	1.21	(1.03, 1.42)	1.19	(1.00, 1.42)	0.050
fourth	1.40	(1.19, 1.64)	1.43	(1.20, 1.70)	< 0.001
fifth (highest)	1.20	(0.99, 1.44)	1.38	(1.12, 1.69)	0.002
unknown	1.37	(1.11, 1.70)	1.43	(1.11, 1.84)	0.006
Situational vulnerability (ref = first quintile)^b^	-	-	-	-	< 0.001
second	0.96	(0.82, 1.13)	0.98	(0.82, 1.16)	0.782
third	1.02	(0.87, 1.19)	0.96	(0.81, 1.14)	0.639
fourth	1.06	(0.90, 1.25)	0.99	(0.83, 1.18)	0.902
fifth (highest)	0.78	(0.65, 0.93)	0.68	(0.56, 0.83)	< 0.001
unknown^c^	1.09	(0.89, 1.33)	-	-	-
Pre-existing chronic health symptoms					
fatigue	1.98	(1.70, 2.29)	1.22	(1.00, 1.48)	0.047
loss of appetite	3.08	(1.94, 4.89)	1.82	(1.12, 2.94)	0.016
Number of pre-existing chronic health symptoms (ref = 0)	-	-	-	-	< 0.001
1 or 2	1.29	(1.11, 1.47)	1.21	(1.06, 1.38)	0.005
3	1.74	(1.43, 2.11)	1.56	(1.26, 1.93)	< 0.001
> 3	2.21	(1.92, 2.54)	1.47	(1.20, 1.81)	< 0.001
Months since last vaccine dose prior to the month of infection (ref = 3 months or less)	-	-	-	-	< 0.001
not vaccinated	1.96	(1.67, 2.30)	1.54	(1.19, 2.00)	0.001
4–6 months	1.23	(1.08, 1.40)	1.31	(1.15, 1.50)	< 0.001
> 6 months	1.22	(1.02, 1.46)	1.27	(1.05, 1.52)	0.013
unknown	1.11	(0.76, 1.61)	1.01	(0.71, 1.44)	0.960
Time period of first infection (ref = pre-omicron)	-	-	-	-	0.005
Omicron (December 1, 2021 – date of electronic questionnaire completion)	0.57	(0.50, 0.66)	0.70	(0.55, 0.90)	0.005
unknown	1.14	(0.42, 3.11)	1.83	(0.62, 5.36)	0.271
Household member testing positive for SARS-CoV-2 infection (ref = no)	-	-	-	-	< 0.001
yes	0.76	(0.68, 0.85)	0.83	(0.74, 0.94)	0.002
unknown	1.42	(0.73, 2.77)	1.71	(0.88, 3.34)	0.116

Notes: Estimates for Canada exclude the territories. All estimates are weighted. The reference category for a pre-existing chronic condition or symptom are adults without the chronic condition or symptom, respectively

Abbreviations: aOR = adjusted odds ratio, CI = confidence interval, n = unweighted number of respondents included in the final model, ref = reference, uOR = unadjusted odds ratio

*p value is only for the fully adjusted model

^a^ Ethno-cultural composition quantifies a neighbourhood’s makeup of immigrant populations

^b^ Situational vulnerability quantifies a neighbourhood’s education level, Indigenous composition, and extent of dwellings in need of major repairs

^c^ For the dimensions of deprivation, values for an individual were either known or unknown for all dimensions. Consequently, the parameter for unknown situational vulnerability has a value of 0 in the final adjusted model because it is a linear combination of the parameter for unknown ethnocultural composition

With respect to retained covariates, being female, unvaccinated or vaccinated more than 3 months prior to infection, having a pre-existing CHS or specifically reporting pre-existing fatigue or loss of appetite, and living in a more ethnically diverse neighbourhood were associated with higher odds of more severe infection compared to the respective reference groups. Being 65 years old or older at infection or East or Southeast Asian, getting infected on or after December 1st, 2021, having a household member who tested positive for SARS-CoV-2 infection, and residing in a neighbourhood classified in the highest quintile of situational vulnerability were associated with a lower odds of more severe infection compared to the respective reference groups.

In the first sensitivity analysis, restricting the modelling to respondents with a confirmed SARS-CoV-2 infection resulted in some changes (Table 3). For CCs, arthritis (aOR: 1.11; 95% CI: 0.84, 1.42) was no longer significant, whereas having a pre-existing mental health condition was associated with a higher odds of more severe infection (aOR: 1.32; 95% CI: 1.05, 1.66). For covariates, ethnicity, time since last vaccination prior to infection, and pre-existing chronic fatigue were no longer significant, while body mass index, sadness/pessimism/hopelessness/depression (SPHD) (aOR: 0.71, 95% CI: 0.53, 0.95), and swelling (aOR: 1.85, 95% CI: 1.23, 2.79) became significant. Remoteness index also became significant with those living in remote or very remote communities having a significantly lower odds of more severe infection (aOR: 0.31; 95% CI: 0.13, 0.74). Sex significantly interacted with BMI: for males, excess body weight was associated with a higher odds of more severe infection while no association was noted for females. When examined by BMI category, the interaction indicated that the relationship between sex and severity of infection decreased in magnitude with increases in BMI: for underweight or normal weight (aOR: 1.64, 95% CI: 1.29, 2.10) and overweight adults (aOR: 1.24, 95% CI: 1.01, 1.54), females had a higher odds of more severe infection, but this relationship no longer existed among obese adults (aOR: 0.96, 95% CI: 0.75, 1.22).

**Table 3:** Odds ratios for more severe acute first infection symptoms among adults with a confirmed SARS-CoV-2 infection, Canada, January 2020 to August 2022 (*n* = 7092)

Characteristics	uOR	95% CI	aOR	95% CI	*p* value*
Pre-existing chronic conditions					
chronic lung disease	2.48	(1.52, 4.03)	1.98	(1.17, 3.34)	0.011
sleep apnea	1.38	(1.08, 1.76)	1.04	(0.79, 1.37)	0.768
asthma	1.46	(1.15, 1.84)	1.13	(0.89, 1.43)	0.310
chronic heart disease	1.26	(0.88, 1.80)	0.87	(0.57, 1.32)	0.503
diabetes	0.98	(0.72, 1.33)	0.79	(0.57, 1.11)	0.177
chronic kidney disease	1.29	(0.76, 2.19)	1.09	(0.58, 2.07)	0.782
high blood pressure	1.3	(1.09, 1.56)	1.24	(1.02, 1.52)	0.033
chronic blood disorder	1.54	(0.72, 3.28)	0.96	(0.40, 2.33)	0.929
osteoporosis	1.08	(0.67, 1.73)	0.63	(0.40, 1.00)	0.050
back problems	1.48	(1.19, 1.83)	1.05	(0.82, 1.35)	0.715
urinary incontinence	1.24	(0.84, 1.81)	0.94	(0.63, 1.39)	0.750
bowel disorder	1.45	(1.06, 2.00)	0.92	(0.66, 1.29)	0.642
weakened immune system	2.34	(1.66, 3.31)	1.71	(1.20, 2.44)	0.003
chronic neurological disorder	1.17	(0.68, 2.02)	0.72	(0.41, 1.26)	0.245
chronic fatigue syndrome or fibromyalgia	3.75	(2.20, 6.40)	2.11	(1.19, 3.75)	0.010
stroke	0.96	(0.49, 1.85)	0.66	(0.33, 1.31)	0.232
mental health condition	1.68	(1.37, 2.07)	1.32	(1.05, 1.66)	0.019
arthritis	1.54	(1.28, 1.86)	1.11	(0.87, 1.42)	0.396
Age at infection (ref = 15–34)	-	-	-	-	< 0.001
35–49	0.99	(0.86, 1.15)	0.93	(0.80, 1.08)	0.331
50–64	1.05	(0.89, 1.23)	0.90	(0.75, 1.08)	0.265
65+	0.76	(0.64, 0.91)	0.59	(0.47, 0.74)	< 0.001
Sex (ref = male)	-	-	-	-	-
female	1.35	(1.19, 1.52)	Interaction	-	-
Remoteness index (ref = easily accessible area)	-	-	-	-	0.004
accessible area	0.90	(0.78, 1.03)	0.99	(0.85, 1.15)	0.843
less accessible area	1.05	(0.83, 1.34)	1.28	(0.99, 1.67)	0.064
remote or very remote areas	0.26	(0.12, 0.54)	0.31	(0.13, 0.74)	0.008
unknown	0.53	(0.28, 0.99)	0.47	(0.24, 0.91)	0.026
Ethno-cultural composition (ref = first quintile)^a^	-	-	-	-	< 0.001
second	1.35	(1.11, 1.63)	1.34	(1.09, 1.64)	0.005
third	1.28	(1.08, 1.53)	1.34	(1.10, 1.63)	0.003
fourth	1.53	(1.28, 1.83)	1.60	(1.31, 1.96)	< 0.001
fifth (highest)	1.46	(1.19, 1.80)	1.58	(1.26, 1.98)	< 0.001
unknown	1.49	(1.18, 1.89)	1.55	(1.15, 2.09)	0.004
Situational vulnerability (ref = first quintile)^b^	-	-	-	-	0.007
second	0.93	(0.77, 1.12)	0.97	(0.80, 1.18)	0.760
third	0.98	(0.82, 1.18)	0.95	(0.78, 1.15)	0.599
fourth	1.14	(0.94, 1.38)	1.07	(0.87, 1.31)	0.540
fifth (highest)	0.75	(0.61, 0.93)	0.69	(0.55, 0.87)	0.002
unknown^c^	1.08	(0.86, 1.36)	-	-	-
Body mass index (BMI) (ref = underweight and normal weight (BMI < 25 kg/m2))	-	-	-	-	-
overweight (25 kg/m2 < = BMI < 30 kg/m2)	1.07	(0.92, 1.24)	Interaction	-	-
obese (BMI > = 30 kg/m2)	1.38	(1.18, 1.61)	Interaction	-	-
unknown	1.36	(0.85, 2.20)	Interaction	-	-
Pre-existing chronic health symptoms					
loss of appetite	3.51	(1.90, 6.47)	2.03	(1.07, 3.85)	0.030
swelling	3.21	(2.13, 4.84)	1.85	(1.23, 2.79)	0.003
sadness/pessimism/hopelessness/depression	1.44	(1.13, 1.83)	0.71	(0.53, 0.95)	0.021
Number of pre-existing chronic health symptoms (ref = 0)	-	-	-	-	< 0.001
1 or 2	1.34	(1.16, 1.55)	1.32	(1.14, 1.53)	< 0.001
3	1.78	(1.42, 2.22)	1.83	(1.44, 2.33)	< 0.001
> 3	2.29	(1.93, 2.72)	1.99	(1.59, 2.50)	< 0.001
Time period of first infection (ref = pre-omicron)	-	-	-	-	0.002
Omicron (December 1, 2021 – date of electronic questionnaire completion)	0.72	(0.60, 0.87)	0.70	(0.58, 0.86)	0.001
unknown	0.77	(0.18, 3.30)	1.03	(0.20, 5.20)	0.972
Household member testing positive for SARS-CoV-2 infection (ref = no)	-	-	-	-	0.038
yes	0.92	(0.80, 1.05)	0.94	(0.81, 1.09)	0.398
unknown	2.09	(0.95, 4.58)	2.22	(1.08, 4.53)	0.029
Interaction between sex and BMI	-	-	-	-	0.017
male (ref = underweight or normal weight)	-	-	-	-	-
overweight	-	-	1.36	(1.06, 1.75)	0.014
obese	-	-	1.79	(1.36, 2.36)	< 0.001
unknown			1.62	(0.06, 42.38)	0.772
female (ref = underweight or normal weight)	-	-	-	-	-
overweight	-	-	1.03	(0.84, 1.27)	0.758
obese	-	-	1.05	(0.85, 1.28)	0.670
unknown			1.05	(0.66, 1.69)	0.834
female underweight or normal weight (ref = male underweight or normal weight)	-	-	1.64	(1.29, 2.10)	< 0.001
female overweight (ref = male overweight)	-	-	1.24	(1.01, 1.54)	0.045
female obese (ref = male obese)	-	-	0.96	(0.75, 1.22)	0.717
female unknown (ref = male unknown)	-	-	1.07	(0.04, 27.51)	0.969

Notes: Estimates for Canada exclude the territories. All estimates are weighted. The reference category for a pre-existing chronic condition or symptom are adults without the chronic condition or symptom, respectively

Abbreviations: aOR = adjusted odds ratio, CI = confidence interval, n = unweighted number of respondents included in the final model, ref = reference, uOR = unadjusted odds ratio

*p value is only for the fully adjusted model

^a^ Ethno-cultural composition quantifies a neighbourhood’s makeup of immigrant populations

^b^ Situational vulnerability quantifies a neighbourhood’s education level, Indigenous composition, and extent of dwellings in need of major repairs

^c^ For the dimensions of deprivation, values for an individual were either known or unknown for all dimensions. Consequently, the parameter for unknown situational vulnerability has a value of 0 in the final adjusted model because it is a linear combination of the parameter for unknown ethnocultural composition

When recoding the CCs as trichotomous variables to account for duration of chronic conditions prior to infection, the majority of the model remained the same (compare Table 2 with Table 4). Among the significant CCs, CLC, HBP, osteoporosis, and WIS had significant associations when the CC was diagnosed 10 or more years prior to infection. Only HBP and CFS had significant associations when the CC was diagnosed less than 10 years prior to infection. Notably, arthritis was no longer statistically significant and an interaction between bowel disorder and sex was observed. For males, there was no significant association between bowel disorders and severity of infection, but females diagnosed 10 or more years prior to infection had higher odds of a more severe infection than females without bowel disorders (aOR: 1.78, 95% CI: 1.15, 2.74). When examined by bowel disorders category, the odds of a more severe infection were higher for females than males among adults without bowel disorders (aOR: 1.29, 95% CI: 1.14, 1.45) or with bowel disorders diagnosed 10 or more years prior to infection (aOR: 4.16, 95% CI: 1.62, 10.68), but not among adults with bowel disorders diagnosed less than 10 years prior to infection (aOR: 1.06, 95% CI: 0.51, 2.18). For covariates, only fatigue was no longer statistically significant.

**Table 4:** Odds ratios for more severe acute first infection symptoms among adults with a self-reported confirmed or suspected SARS-CoV-2 infection while adjusting for duration of pre-existing chronic conditions, Canada, January 2020 to August 2022 (*n* = 8991)

Characteristics	uOR	95% CI	aOR	95% CI	*p* value*
Pre-existing chronic conditions					
chronic lung disease					0.038
diagnosed less than 10 years prior to infection	1.94	(1.12, 3.38)	1.54	(0.82, 2.88)	0.181
diagnosed 10 or more years prior to infection	3.01	(1.82, 4.99)	1.91	(1.08, 3.38)	0.027
sleep apnea					0.196
diagnosed less than 10 years prior to infection	1.46	(1.16, 1.84)	1.27	(0.98, 1.65)	0.072
diagnosed 10 or more years prior to infection	1.31	(0.86, 2.00)	1.00	(0.63, 1.58)	0.992
asthma					0.280
diagnosed less than 10 years prior to infection	1.86	(1.13, 3.05)	1.29	(0.77, 2.16)	0.339
diagnosed 10 or more years prior to infection	1.56	(1.24, 1.96)	1.17	(0.92, 1.49)	0.196
chronic heart disease					0.305
diagnosed less than 10 years prior to infection	1.33	(0.84, 2.11)	1.04	(0.62, 1.73)	0.888
diagnosed 10 or more years prior to infection	0.98	(0.60, 1.60)	0.68	(0.41, 1.12)	0.132
diabetes					0.392
diagnosed less than 10 years prior to infection	1.24	(0.83, 1.85)	0.98	(0.64, 1.50)	0.920
diagnosed 10 or more years prior to infection	0.94	(0.65, 1.36)	0.75	(0.50, 1.13)	0.172
chronic kidney disease					0.990
diagnosed less than 10 years prior to infection	1.25	(0.65, 2.43)	1.06	(0.48, 2.30)	0.893
diagnosed 10 or more years prior to infection	0.97	(0.50, 1.91)	0.98	(0.40, 2.43)	0.967
high blood pressure					0.007
diagnosed less than 10 years prior to infection	1.45	(1.16, 1.80)	1.32	(1.05, 1.67)	0.018
diagnosed 10 or more years prior to infection	1.27	(1.03, 1.57)	1.35	(1.06, 1.71)	0.016
chronic blood disorder					0.416
diagnosed less than 10 years prior to infection	1.75	(0.57, 5.36)	0.94	(0.26, 3.45)	0.928
diagnosed 10 or more years prior to infection	0.91	(0.36, 2.33)	0.50	(0.18, 1.41)	0.187
osteoporosis					0.029
diagnosed less than 10 years prior to infection	1.46	(0.85, 2.50)	0.67	(0.39, 1.15)	0.144
diagnosed 10 or more years prior to infection	0.81	(0.45, 1.47)	0.48	(0.26, 0.89)	0.021
back problems					0.246
diagnosed less than 10 years prior to infection	1.37	(1.02, 1.83)	0.99	(0.72, 1.37)	0.951
diagnosed 10 or more years prior to infection	1.86	(1.50, 2.30)	1.23	(0.96, 1.56)	0.097
urinary incontinence					0.356
diagnosed less than 10 years prior to infection	1.55	(1.06, 2.26)	1.06	(0.70, 1.60)	0.792
diagnosed 10 or more years prior to infection	0.94	(0.50, 1.79)	0.61	(0.31, 1.21)	0.157
bowel disorder			-	-	-
diagnosed less than 10 years prior to infection	1.43	(1.02, 2.00)	Interaction	-	-
diagnosed 10 or more years prior to infection	2.05	(1.32, 3.16)	Interaction	-	-
weakened immune system					0.037
diagnosed less than 10 years prior to infection	2.03	(1.37, 2.99)	1.43	(0.93, 2.21)	0.105
diagnosed 10 or more years prior to infection	2.96	(1.93, 4.55)	1.57	(1.03, 2.38)	0.034
chronic neurological disorder					0.732
diagnosed less than 10 years prior to infection	1.45	(0.75, 2.81)	0.82	(0.44, 1.49)	0.507
diagnosed 10 or more years prior to infection	2.02	(1.09, 3.76)	1.16	(0.57, 2.34)	0.689
chronic fatigue syndrome or fibromyalgia					0.020
diagnosed less than 10 years prior to infection	4.09	(2.28, 7.35)	2.13	(1.10, 4.12)	0.024
diagnosed 10 or more years prior to infection	5.05	(2.83, 8.98)	1.83	(0.98, 3.42)	0.058
stroke					0.858
diagnosed less than 10 years prior to infection	0.97	(0.49, 1.94)	0.82	(0.35, 1.91)	0.643
diagnosed 10 or more years prior to infection	1.32	(0.36, 4.82)	0.81	(0.21, 3.13)	0.763
mental health condition					0.276
diagnosed less than 10 years prior to infection	1.44	(1.12, 1.86)	1.02	(0.79, 1.33)	0.857
diagnosed 10 or more years prior to infection	1.90	(1.56, 2.31)	1.20	(0.96, 1.50)	0.109
arthritis					0.156
diagnosed less than 10 years prior to infection	1.69	(1.32, 2.17)	1.22	(0.91, 1.64)	0.180
diagnosed 10 or more years prior to infection	1.70	(1.40, 2.08)	1.22	(0.96, 1.55)	0.097
Age at infection (ref = 15–34)	-	-	-	-	< 0.001
35–49	1.02	(0.90, 1.16)	1.04	(0.91, 1.19)	0.568
50–64	1.10	(0.96, 1.26)	0.94	(0.81, 1.10)	0.429
65+	0.87	(0.74, 1.02)	0.68	(0.55, 0.83)	< 0.001
Sex (ref = male)	-	-	-	-	-
female	1.44	(1.29, 1.60)	Interaction	-	-
Ethnicity (ref = white)	-	-	-	-	0.016
Indigenous	1.26	(0.97, 1.64)	1.13	(0.85, 1.50)	0.409
South Asian	0.97	(0.71, 1.32)	0.93	(0.66, 1.30)	0.653
East/Southeast Asian	0.64	(0.51, 0.80)	0.62	(0.48, 0.79)	< 0.001
Black	0.90	(0.57, 1.44)	0.81	(0.50, 1.33)	0.411
Arab/West Asian	0.73	(0.48, 1.10)	0.72	(0.46, 1.13)	0.152
Latin American	0.87	(0.56, 1.34)	0.87	(0.57, 1.34)	0.535
Mixed/Other	1.09	(0.80, 1.47)	1.10	(0.80, 1.52)	0.554
Ethno-cultural composition (ref = first quintile)^a^	-	-	-	-	< 0.001
second	1.27	(1.07, 1.51)	1.23	(1.02, 1.49)	0.029
third	1.21	(1.03, 1.42)	1.19	(1.00, 1.42)	0.051
fourth	1.40	(1.19, 1.64)	1.43	(1.20, 1.71)	< 0.001
fifth (highest)	1.20	(0.99, 1.44)	1.41	(1.15, 1.73)	0.001
unknown	1.37	(1.11, 1.70)	1.46	(1.13, 1.88)	0.004
Situational vulnerability (ref = first quintile)^b^	-	-	-	-	0.001
second	0.96	(0.82, 1.13)	0.98	(0.83, 1.17)	0.825
third	1.02	(0.87, 1.19)	0.95	(0.80, 1.12)	0.51
fourth	1.06	(0.90, 1.25)	1.00	(0.84, 1.20)	0.996
fifth (highest)	0.78	(0.65, 0.93)	0.68	(0.56, 0.83)	< 0.001
unknown^c^	1.09	(0.89, 1.33)	-	-	-
Pre-existing chronic health symptoms					
loss of appetite	3.08	(1.94, 4.89)	1.96	(1.21, 3.17)	0.006
Number of pre-existing chronic health symptoms (ref = 0)	-	-	-	-	< 0.001
1 or 2	1.29	(1.13, 1.47)	1.23	(1.08, 1.41)	0.003
3	1.74	(1.43, 2.11)	1.68	(1.36, 2.07)	< 0.001
> 3	2.21	(1.92, 2.54)	1.66	(1.38, 1.99)	< 0.001
Months since last vaccine dose prior to the month of infection (ref = 3 months or less)	-	-	-	-	< 0.001
not vaccinated	1.95	(1.66, 2.29)	1.47	(1.13, 1.93)	0.005
4–6 months	1.23	(1.08, 1.40)	1.32	(1.16, 1.51)	< 0.001
> 6 months	1.22	(1.02, 1.46)	1.28	(1.06, 1.54)	0.009
unknown	1.11	(0.76, 1.61)	1.11	(0.77, 1.62)	0.571
Time period of first infection (ref = pre-omicron)	-	-	-	-	0.007
Omicron (December 1, 2021 – date of electronic questionnaire completion)	0.57	(0.50, 0.66)	0.67	(0.52, 0.86)	0.002
unknown	1.14	(0.42, 3.11)	1.30	(0.04, 47.38)	0.885
Household member testing positive for SARS-CoV-2 infection (ref = no)	-	-	-	-	0.001
yes	0.76	(0.68, 0.85)	0.84	(0.75, 0.95)	0.005
unknown	1.42	(0.73, 2.77)	1.79	(0.91, 3.52)	0.094
Interaction between sex and bowel disorder	-	-	-	-	0.043
Male (ref = no bowel disorder)	-	-	-	-	-
bowel disorder diagnosed less than 10 years prior to infection	-	-	1.06	(0.60, 1.88)	0.840
bowel disorder diagnosed 10 or more years prior to infection	-	-	0.55	(0.24, 1.28)	0.166
Female (ref = no bowel disorder)	-	-	-	-	-
bowel disorder diagnosed less than 10 years prior to infection	-	-	0.87	(0.55, 1.39)	0.567
bowel disorder diagnosed 10 or more years prior to infection	-	-	1.78	(1.15, 2.74)	0.009
Female no bowel disorder (ref = male no bowel disorder)	-	-	1.29	(1.14, 1.45)	< 0.001
Female bowel disorder diagnosed less than 10 years prior to infection (ref = male bowel disorder diagnosed less than 10 years prior to infection)	-	-	1.06	(0.51, 2.18)	0.881
Female bowel disorder diagnosed 10 or more years prior to infection (ref = male bowel disorder diagnosed 10 or more years prior to infection)	-	-	4.16	(1.62, 10.68)	0.003

Notes: Estimates for Canada exclude the territories. All estimates are weighted. The reference category for a pre-existing chronic condition or symptom are adults without the chronic condition or symptom, respectively

Abbreviations: aOR = adjusted odds ratio, CI = confidence interval, n = unweighted number of respondents included in the final model, ref = reference, uOR = unadjusted odds ratio

*p value is only for the fully adjusted model

^a^ Ethno-cultural composition quantifies a neighbourhood’s makeup of immigrant populations

^b^ Situational vulnerability quantifies a neighbourhood’s education level, Indigenous composition, and extent of dwellings in need of major repairs

^c^ For the dimensions of deprivation, values for an individual were either known or unknown for all dimensions. Consequently, the parameter for unknown situational vulnerability has a value of 0 in the final adjusted model because it is a linear combination of the parameter for unknown ethnocultural composition

After dichotomizing the severity of infection variable, CLC, HBP, osteoporosis, WIS, and arthritis were no longer significant. However, CFS remained significant (aOR: 2.09, 95% CI: 1.23, 3.53) and chronic kidney disease (CKD) emerged as a newly significant CC (aOR: 0.38, 95% CI: 0.15, 0.92) (Table 5). For covariates, ethnicity, situational vulnerability, household member testing positive for SARS-CoV-2 infection, and pre-existing fatigue were no longer significant. Region of residence became significant with the Prairies being the only region showing a significant difference when compared to Ontario (aOR: 0.75, 95% CI: 0.60, 0.94). Also, the number of pre-existing CCs became significant: the odds of a more severe infection were 1.72 (95% CI: 1.13, 2.61) times greater among adults with 2 CCs compared to adults with none of the CCs examined. As observed in the first sensitivity analysis, sex significantly interacted with BMI and followed the same patterns seen in Table 3.

**Table 5:** Odds ratios for severe^a^ acute first infection symptoms among adults with a self-reported confirmed or suspected SARS-CoV-2 infection, Canada, January 2020 to August 2022 (*n* = 9107)

Characteristics	uOR	95% CI	aOR	95% CI	*p* value*
Pre-existing chronic conditions					
chronic lung disease	2.92	(2.02, 4.20)	1.55	(0.97, 2.46)	0.064
sleep apnea	1.57	(1.21, 2.03)	0.92	(0.65, 1.30)	0.631
asthma	1.83	(1.44, 2.33)	1.12	(0.83, 1.50)	0.469
chronic heart disease	1.43	(0.93, 2.20)	0.82	(0.50, 1.35)	0.441
diabetes	1.75	(1.34, 2.28)	1.15	(0.84, 1.59)	0.383
chronic kidney disease	0.65	(0.31, 1.38)	0.38	(0.15, 0.92)	0.032
high blood pressure	1.70	(1.40, 2.06)	1.17	(0.88, 1.55)	0.275
chronic blood disorder	1.74	(0.87, 3.46)	0.80	(0.34, 1.88)	0.609
osteoporosis	1.68	(1.13, 2.49)	0.83	(0.51, 1.34)	0.443
back problems	2.10	(1.73, 2.55)	1.20	(0.88, 1.63)	0.247
urinary incontinence	1.42	(0.94, 2.16)	0.74	(0.47, 1.18)	0.211
bowel disorder	2.20	(1.66, 2.92)	1.17	(0.83, 1.64)	0.374
weakened immune system	2.62	(1.93, 3.56)	1.40	(0.94, 2.10)	0.098
chronic neurological disorder	2.23	(1.44, 3.44)	1.11	(0.63, 1.93)	0.724
chronic fatigue syndrome or fibromyalgia	4.88	(3.18, 7.47)	2.09	(1.23, 3.53)	0.006
stroke	1.20	(0.57, 2.50)	0.56	(0.25, 1.25)	0.157
mental health condition	1.93	(1.61, 2.32)	1.15	(0.86, 1.53)	0.343
arthritis	2.13	(1.77, 2.57)	1.14	(0.84, 1.54)	0.402
Age at infection (ref = 15–34)	-	-	-	-	0.481
35–49	1.11	(0.92, 1.33)	1.06	(0.87, 1.29)	0.593
50–64	1.29	(1.06, 1.56)	0.98	(0.78, 1.23)	0.842
65+	1.18	(0.94, 1.48)	0.85	(0.62, 1.15)	0.280
Sex (ref = male)	-	-	-	-	-
female	1.33	(1.16, 1.56)	Interaction	-	-
Number of pre-existing chronic health conditions (ref = 0)	-	-	-	-	0.013
1	1.35	(1.11, 1.63)	1.10	(0.85, 1.42)	0.475
2	2.62	(2.10, 3.28)	1.72	(1.13, 2.61)	0.011
3	2.50	(1.92, 3.27)	1.34	(0.76, 2.35)	0.314
> 3	3.48	(2.74, 4.43)	1.28	(0.55, 2.98)	0.565
Ethno-cultural composition (ref = first quintile)^b^	-	-	-	-	0.014
second	1.38	(1.08, 1.75)	1.32	(1.02, 1.71)	0.036
third	1.12	(0.89, 1.41)	1.11	(0.85, 1.44)	0.435
fourth	1.16	(0.92, 1.47)	1.26	(0.97, 1.63)	0.089
fifth (highest)	1.27	(0.97, 1.65)	1.35	(1.00, 1.82)	0.047
unknown	1.47	(1.11, 1.95)	1.71	(1.25, 2.35)	0.001
Region of residence (ref = Ontario)	-	-	-	-	0.012
British Columbia	1.14	(0.92, 1.41)	1.10	(0.88, 1.39)	0.408
Prairies	0.89	(0.73, 1.09)	0.75	(0.60, 0.94)	0.011
Quebec	0.77	(0.63, 0.94)	0.89	(0.71, 1.11)	0.289
Atlantic Canada	0.95	(0.78, 1.15)	0.97	(0.77, 1.21)	0.768
Body mass index (BMI) (ref = underweight and normal weight (BMI < 25 kg/m2))	-	-	-	-	-
overweight (25 kg/m2 < = BMI < 30 kg/m2)	1.00	(0.83, 1.21)	Interaction	-	-
obese (BMI > = 30 kg/m2)	1.63	(1.35, 1.98)	Interaction	-	-
unknown	1.22	(0.69, 2.18)	Interaction	-	-
Pre-existing chronic health symptoms					
loss of appetite	3.63	(2.33, 5.63)	2.13	(1.31, 3.46)	0.002
Number of pre-existing chronic health symptoms (ref = 0)	-	-	-	-	< 0.001
1 or 2	1.51	(1.25, 1.83)	1.37	(1.12, 1.67)	0.002
3	1.87	(1.44, 2.43)	1.62	(1.21, 2.16)	0.001
> 3	2.86	(2.39, 3.43)	1.87	(1.45, 2.39)	< 0.001
Months since last vaccine dose prior to the month of infection (ref= <=3 months)					< 0.001
Not vaccinated	2.73	(2.22, 3.34)	1.76	(1.31, 2.37)	< 0.001
4–6 months	1.19	(0.96, 1.47)	1.29	(1.04, 1.61)	0.019
> 6 months	1.52	(1.17, 1.97)	1.67	(1.27, 2.19)	< 0.001
Unknown	1.60	(0.95, 2.71)	1.28	(0.75, 2.19)	0.362
Time period of first infection (ref = pre-omicron)	-	-	-	-	< 0.001
Omicron (December 1, 2021 – date of electronic questionnaire completion)	0.41	(0.35, 0.48)	0.50	(0.38, 0.66)	< 0.001
unknown	1.48	(0.56, 3.87)	1.95	(0.68, 5.54)	0.212
Interaction between sex and BMI	-	-	-	-	0.003
male (ref = underweight or normal weight)	-	-	-	-	-
overweight	-	-	1.27	(0.89, 1.81)	0.183
obese	-	-	2.21	(1.54, 3.18)	< 0.001
unknown			2.88	(0.15, 53.63)	0.478
female (ref = underweight or normal weight)	-	-	-	-	-
overweight	-	-	0.92	(0.71, 1.21)	0.562
obese	-	-	1.02	(0.80, 1.31)	0.848
unknown			0.73	(0.38, 1.40)	0.346
female underweight or normal weight (ref = male underweight or normal weight)	-	-	1.81	(1.27, 2.56)	0.001
female overweight (ref = male overweight)	-	-	1.31	(1.00, 1.72)	0.051
female obese (ref = male obese)	-	-	0.84	(0.64, 1.09)	0.180
female unknown (ref = male unknown)	-	-	0.46	(0.02, 8.96)	0.607

Notes: Estimates for Canada exclude the territories. All estimates are weighted. The reference category for a pre-existing chronic condition or symptom are adults without the chronic condition or symptom, respectively

Abbreviations: aOR = adjusted odds ratio, CI = confidence interval, n = unweighted number of respondents included in the final model, ref = reference, uOR = unadjusted odds ratio

*p value is only for the fully adjusted model

^a^ Severe symptoms were defined as significantly affecting daily life or requiring hospitalization

^b^ Ethno-cultural composition quantifies a neighbourhood’s makeup of immigrant populations

Excluding individuals with missing chronic condition diagnosis dates resulted in little changes when compared to Table 2 (Table 6). Only pre-existing fatigue was no longer significant, while pre-existing symptoms relating to the heart became significant.

**Table 6:** Odds ratios for more severe acute first infection symptoms among adults with a self-reported confirmed or suspected SARS-CoV-2 infection, Canada, January 2020 to August 2022 (excludes respondents with missing chronic condition diagnosis dates) (*n* = 9026)

Characteristics	uOR	95% CI	aOR	95% CI	*p* value*
Pre-existing chronic conditions					
chronic lung disease	2.39	(1.65, 3.45)	1.64	(1.08, 2.47)	0.020
sleep apnea	1.42	(1.14, 1.76)	1.18	(0.93, 1.49)	0.180
asthma	1.59	(1.30, 1.96)	1.22	(0.98, 1.52)	0.075
chronic heart disease	1.16	(0.83, 1.64)	0.76	(0.52, 1.12)	0.166
diabetes	1.12	(0.85, 1.46)	0.89	(0.66, 1.19)	0.431
chronic kidney disease	1.13	(0.71, 1.80)	0.96	(0.54, 1.69)	0.882
high blood pressure	1.36	(1.17, 1.60)	1.32	(1.11, 1.58)	0.002
chronic blood disorder	1.19	(0.59, 2.40)	0.65	(0.30, 1.43)	0.286
osteoporosis	1.08	(0.71, 1.63)	0.59	(0.39, 0.89)	0.012
back problems	1.63	(1.37, 1.95)	1.12	(0.92, 1.38)	0.266
urinary incontinence	1.30	(0.94, 1.80)	0.90	(0.63, 1.26)	0.525
bowel disorder	1.68	(1.27, 2.23)	1.08	(0.82, 1.42)	0.599
weakened immune system	2.37	(1.77, 3.16)	1.48	(1.08, 2.02)	0.014
chronic neurological disorder	1.71	(1.09, 2.70)	0.93	(0.59, 1.48)	0.760
chronic fatigue syndrome or fibromyalgia	4.57	(3.00, 6.97)	2.12	(1.34, 3.36)	0.001
stroke	1.06	(0.58, 1.94)	0.78	(0.40, 1.51)	0.454
mental health condition	1.64	(1.39, 1.94)	1.10	(0.91, 1.32)	0.326
arthritis	1.70	(1.44, 2.00)	1.24	(1.01, 1.52)	0.044
Age at infection (ref = 15–34)	-	-	-	-	< 0.001
35–49	1.02	(0.90, 1.16)	1.04	(0.91, 1.19)	0.559
50–64	1.10	(0.96, 1.26)	0.96	(0.83, 1.12)	0.628
65+	0.87	(0.74, 1.02)	0.67	(0.55, 0.83)	< 0.001
Sex (ref = male)	-	-	-	-	< 0.001
female	1.44	(1.29, 1.60)	1.31	(1.16, 1.47)	< 0.001
Ethnicity (ref = white)	-	-	-	-	0.023
Indigenous	1.26	(0.97, 1.64)	1.14	(0.87, 1.50)	0.338
South Asian	0.97	(0.71, 1.32)	0.93	(0.66, 1.29)	0.648
East/Southeast Asian	0.64	(0.51, 0.80)	0.62	(0.49, 0.79)	< 0.001
Black	0.90	(0.57, 1.44)	0.81	(0.50, 1.32)	0.400
Arab/West Asian	0.73	(0.48, 1.10)	0.72	(0.46, 1.13)	0.152
Latin American	0.87	(0.56, 1.34)	0.89	(0.57, 1.37)	0.582
Mixed/Other	1.09	(0.80, 1.47)	1.09	(0.79, 1.51)	0.597
Ethno-cultural composition (ref = first quintile)^a^	-	-	-	-	0.002
second	1.27	(1.07, 1.51)	1.25	(1.04, 1.51)	0.016
third	1.21	(1.03, 1.42)	1.19	(1.00, 1.42)	0.052
fourth	1.40	(1.19, 1.64)	1.43	(1.20, 1.70)	< 0.001
fifth (highest)	1.20	(0.99, 1.44)	1.39	(1.13, 1.71)	0.002
unknown	1.37	(1.11, 1.70)	1.44	(1.12, 1.87)	0.005
Situational vulnerability (ref = first quintile)^b^	-	-	-	-	0.001
second	0.96	(0.82, 1.13)	0.97	(0.82, 1.15)	0.716
third	1.02	(0.87, 1.19)	0.95	(0.80, 1.12)	0.517
fourth	1.06	(0.90, 1.25)	0.98	(0.82, 1.17)	0.851
fifth (highest)	0.78	(0.65, 0.93)	0.68	(0.56, 0.82)	< 0.001
unknown^c^	1.09	(0.89, 1.33)	-	-	-
Pre-existing chronic health symptoms					
symptoms relating to the heart	1.94	(1.53, 2.47)	1.33	(1.01, 1.75)	0.039
loss of appetite	3.08	(1.94, 4.89)	1.80	(1.11, 2.92)	0.017
Number of pre-existing chronic health symptoms (ref = 0)	-	-	-	-	< 0.001
1 or 2	1.29	(1.11, 1.47)	1.21	(1.06, 1.39)	0.005
3	1.74	(1.43, 2.11)	1.64	(1.33, 2.03)	< 0.001
> 3	2.21	(1.92, 2.54)	1.60	(1.32, 1.93)	< 0.001
Months since last vaccine dose prior to the month of infection (ref = 3 months or less)	-	-	-	-	< 0.001
not vaccinated	1.96	(1.67, 2.30)	1.49	(1.14, 1.93)	0.003
4–6 months	1.23	(1.08, 1.40)	1.33	(1.16, 1.51)	< 0.001
> 6 months	1.22	(1.02, 1.46)	1.28	(1.06, 1.54)	0.009
unknown	1.11	(0.76, 1.61)	1.04	(0.72, 1.50)	0.822
Time period of first infection (ref = pre-omicron)	-	-	-	-	0.004
Omicron (December 1, 2021 – date of electronic questionnaire completion)	0.57	(0.50, 0.66)	0.67	(0.53, 0.87)	0.002
unknown	1.14	(0.42, 3.11)	1.48	(0.50, 4.37)	0.482
Household member testing positive for SARS-CoV-2 infection (ref = no)	-	-	-	-	0.001
yes	0.76	(0.68, 0.85)	0.84	(0.74, 0.94)	0.004
unknown	1.42	(0.73, 2.77)	1.77	(0.90, 3.49)	0.099

Notes: Estimates for Canada exclude the territories. All estimates are weighted. The reference category for a pre-existing chronic condition or symptom are adults without the chronic condition or symptom, respectively

Abbreviations: aOR = adjusted odds ratio, CI = confidence interval, n = unweighted number of respondents included in the final model, ref = reference, uOR = unadjusted odds ratio

*p value is only for the fully adjusted model

^a^ Ethno-cultural composition quantifies a neighbourhood’s makeup of immigrant populations

^b^ Situational vulnerability quantifies a neighbourhood’s education level, Indigenous composition, and extent of dwellings in need of major repairs

^c^ For the dimensions of deprivation, values for an individual were either known or unknown for all dimensions. Consequently, the parameter for unknown situational vulnerability has a value of 0 in the final adjusted model because it is a linear combination of the parameter for unknown ethnocultural composition

## Discussion

We used data from a large population-based Canadian survey to examine relationships between pre-existing CCs and severity of acute SARS-CoV-2 infection. Among adults with a confirmed or suspected SARS-CoV-2 infection, six of the 21 examined CCs were significantly associated with more severe infection as measured by their impact on daily life. When limiting the analyses to confirmed infections, we found that those with pre-existing mental health conditions also had greater odds of more severe infection.

Depending on the CC, our findings deviate from or corroborate the existing evidence, which is mainly based on populations accessing health care services following infection. A systematic review identified strong relationships between COVID-19 severity, defined as mortality or the most severe outcome such as intensive care unit (ICU) admission, and chronic obstructive lung disease (COPD), chronic kidney disease, cardiovascular diseases, hypertension, and diabetes [39]. We also found that CLCs, including COPD, and hypertension were significantly associated with more severe infection. However, we found no relationship for diabetes and chronic kidney disease. For those with a weakened immune system, our findings support the evidence that the immunocompromised subpopulation is at increased risk of severe SARS-CoV-2 infection outcomes [40, 41]. The lack of association between certain CCs and severity of infection could be caused, at least in part, by the correlations between CCs. For example, among adults with confirmed and suspected infections, those with hypertension were about six times more likely to have diabetes (19.6% vs. 3.3%, *p* < 0.0001) and about five times more likely to have chronic kidney disease (2.0% vs.0.4%, *p* < 0.0001) compared to those without hypertension.

A Swedish study found that a SARS-CoV-2 infection can be a potent trigger for reactivation of latent herpes viruses and endogenous retroviruses in those with pre-existing CFS [42]. There is a paucity of research evaluating COVID-19 severity in those with pre-existing fibromyalgia; we identified only one study, which found no association between fibromyalgia and hospitalization for COVID-19^27^. One proposed mechanism is that since fibromyalgia is triggered by mental stress and anxiety, the indirect impact of the COVID-19 pandemic could have triggered a more severe manifestation of fibromyalgia that coincided with an actual infection [43].

Potential contributors to differing results between this study and existing evidence are the source population of participants and methods of measuring severity of infection. Our study did not require a healthcare encounter for eligibility and captured a broader spectrum of severity of infection while most other studies identified participants from those seeking care for their symptoms and focussed on severe outcomes like hospitalization, ICU admission or death. Individuals who seek medical care for COVID-19 are likely having more severe symptoms or perceive their symptoms as severe enough to seek care. Additionally, individuals who have died due to COVID-19 were not captured by CCAHS-2. If this population had been captured, stronger relationships for several CCs and covariates may have been observed [44, 45]. This methodological difference could also explain some of our counterintuitive findings. Specifically, significant protective effects were associated with being male, aged 65 + years at infection, and living in a remote community and/or neighbourhood with high situational vulnerability (i.e., low education level, high Indigenous composition, and high proportion of dwellings in need of major repairs), all characteristics related to a higher risk of mortality from COVID-19 [44,46]. It can also explain why no significant relationship was found for diabetes and CKD, as those living with these CCs have a higher risk of mortality following SARS-CoV-2 infection than those without the respective CC [47, 48].

This limitation may also help explain the observed significant protective effect of osteoporosis. Ahn et al. found that individuals with a history of osteoporosis who contracted a SARS-CoV-2 infection did not experience significant differences in most clinical outcomes compared to those without such a history [49]. However, osteoporosis patients with a history of fracture had an elevated risk of severe complications, while osteoporosis patients without fractures had a lower risk compared to those without osteoporosis. Given that fractures are a common consequence of osteoporosis, and osteoporosis patients with fractures tend to experience high mortality rates regardless of COVID-19 status, excluding this high-risk subpopulation may bias the results toward a protective effect [50, 51].

For the other covariates, our findings support the literature showing more severe infections were associated with pre-Omicron infections [52] and being unvaccinated or not recently vaccinated [53]. The protective effect of having a household member testing positive for SARS-CoV-2 infection could result from being better prepared for the perceived impacts of the infection.

Limiting the modeling to adults with confirmed infections had an impact on some of the variables retained and adjusted associations. This may be due to excluding suspected infections that were unrelated to SARS-CoV-2, as well as clinical differences in patients with confirmed vs. suspected infections. Observed differences may also arise from the exclusion of adults suspecting an infection earlier in the pandemic when testing capacity was limited and health outcomes were worse [52].

Redefining the CC variables to include duration of CC (diagnosed either less than 10 years or 10 or more years prior to infection) resulted in minimal changes to the retained variables and adjusted associations. Sex and pre-existing chronic bowel disorders significantly interacted in their relationship with SARS-CoV-2 infection severity. To our knowledge, this interaction has not been previously identified and may reflect sex differences in the experiences of people with inflammatory bowel disorders. Other research indicates that females experience a worse quality of life and higher psychological distress than males, while males experience more bowel-related surgeries and higher mortality risk than females [54, 55].

When severity was redefined as a binary variable, many of the CCs loss statistical significance. This could be attributed to misclassification of moderate and severe infection symptoms biasing associations toward the null, the addition of number of pre-existing chronic conditions to the model, or the greater importance of other included covariates when examining associations with severe infections.

Excluding individuals with missing chronic condition diagnosis dates from the analytical sample resulted in minimal changes to the final model. Pre-existing fatigue was no longer significant, while pre-existing symptoms relating to the heart became significant. Most odds ratios were unaffected. These results indicate that our approach for handling adults with missing chronic condition diagnosis dates did not introduce bias.

To our knowledge, there is limited research reporting interactions between BMI and sex when examining severity of SARS-CoV-2 infections. A Brazilian study looking at mortality amongst obese individuals hospitalized with COVID-19 found that the more obese a male was, the higher were the odds of mortality, whereas the odds of death among females increased only among those with a BMI of ≥ 50 kg/m^2^ [56]. Additionally, Yamamoto et al. found that higher BMI was associated with lower SARS-CoV-2 spike antibody titers from vaccination in men, but not in females [57]. This suggests that vaccinated males with higher BMI are more at risk for severe COVID-19 outcomes than vaccinated females of comparable BMI which aligns with our findings.

### Strengths and limitations

The primary strength of our study is that it is population-based and considers a wide range of individual characteristics. CCAHS-2 captured dates for SARS-CoV-2 infection, CC diagnosis, chronic health symptom occurrence, and vaccination. Using this data, we were able to determine the temporality of CCs, CHSs and vaccinations in relation to the infection. We also included individuals who suspected they had a SARS-CoV-2 infection but could not access COVID-19 testing or chose not to be tested. This approach increases the applicability of our findings to the general population.

One of the limitations of this study is that those who have died due to COVID-19-related causes were not included. Consequently, the subpopulation who had the most severe SARS-CoV-2 infections were not included in the statistical modelling which could have resulted in lower odds ratio estimates for pre-existing CCs. This effect would be greater for CCs that have established links to higher COVID-19-related mortality. Although the focus of this study was to estimate the impact on daily lives, including the subpopulation who died would have generated more universally interpretable estimates. Another limitation is that a respondent may have been unknowingly infected prior to their first reported SARS-CoV-2 infection. As a result, the severity of their first reported infection could be influenced by CHSs from long COVID. Additional limitations are inherent with survey data, such as selection bias, recall error, lack of objective measures of infection severity, and inaccurate infection status information. While the validity of self-reported data is subject to many biases, it remains a valuable and commonly used tool for assessing a respondent’s subjective experiences. Additionally, rigorous planning and quality assurance were undertaken at all stages of the survey design and conduct to mitigate the impact of these biases [32, 35]. Only 25.3% of adults invited to participate were included in the share file used for analysis. As outlined in the methodology, variables highly correlated with responding to the survey were used to adjust survey weights to minimize non-response bias arising from identified differences between respondents and non-respondents. Although weights were adjusted for non-response and calibrated to reflect the target population using auxiliary information, the potential for biased estimates remains if those who participated and agreed to share their data systematically differed from the target population in ways not corrected through weighting. The low response rate also compromised the study’s power to detect statistically significant associations. Due to limited testing capacity early in the pandemic, we included adults who reported a suspected infection in our main analyses; however, some suspected infections may have been the consequence of conditions or infections unrelated to SARS-CoV-2. Conversely, other respondents may have been unaware of a past SARS-CoV-2 infection or may have inappropriately ascribed COVID-19 symptoms to other conditions or infections. To partly address these issues, we performed sensitivity analyses that limited modeling to adults testing positive for SARS-CoV-2 infection.

## Conclusion

The aim of this study was to characterize the association between pre-existing CCs and SARS-COV-2 infection severity among the Canadian adult population by measuring impacts on daily life. The findings suggest that a greater focus should be placed on those who are immunocompromised or have pre-existing CLC, hypertension, fibromyalgia or CFS, arthritis, or mental health condition. Individuals living with these CCs should be informed of the greater impact a SARS-CoV-2 infection can have on their lives so they can take measures to reduce their risk of infection. Targeted prevention strategies and early interventions in this population can help minimize the impact of infection and the burden on health resource.

## Data Availability

The dataset (Canadian COVID-19 Antibody and Health Survey - Cycle 2) supporting the conclusions of this article is the data is available through Statistics Canada’s Research Data Centres (RDC).

## Abbreviations

aOR: adjusted odds ratio
BD: bowel disease
BMI: body mass index
CC: chronic condition
CCAHS-2: Canadian COVID-19 Antibody and Health Survey
CFS: chronic fatigue syndrome
CHS: chronic health symptom
CI: confidence interval
CKD: chronic kidney disease
CLC: chronic lung condition
COPD: chronic obstructive pulmonary disease
CVD: cardiovascular disease
EQ: electronic questionnaire
HBP: high blood pressure
WIS: weakened immune system
